# Use of geographically weighted logistic regression to quantify spatial variation in the environmental and sociodemographic drivers of leptospirosis in Fiji: a modelling study

**DOI:** 10.1016/S2542-5196(18)30066-4

**Published:** 2018-05

**Authors:** Helen J Mayfield, John H Lowry, Conall H Watson, Mike Kama, Eric J Nilles, Colleen L Lau

**Affiliations:** a Department of Global Health, Research School of Population Health, The Australian National University, Canberra, ACT, Australia; b School of People, Environment and Planning, Massey University, Palmerston North, New Zealand; c School of Geography, Earth Science and Environment, The University of the South Pacific, Suva, Fiji; d Department of Infectious Disease Epidemiology, London School of Hygiene & Tropical Medicine, London, UK; e Fiji Centre for Communicable Disease Control, Ministry of Health and Medical Services, Suva, Fiji; f Division of Pacific Technical Support, World Health Organization, Suva, Fiji; g Children's Health and Environment Program, Child Health Research Centre, The University of Queensland, Brisbane, QLD, Australia

## Abstract

**Background:**

Leptospirosis is a globally important zoonotic disease, with complex exposure pathways that depend on interactions between human beings, animals, and the environment. Major drivers of outbreaks include flooding, urbanisation, poverty, and agricultural intensification. The intensity of these drivers and their relative importance vary between geographical areas; however, non-spatial regression methods are incapable of capturing the spatial variations. This study aimed to explore the use of geographically weighted logistic regression (GWLR) to provide insights into the ecoepidemiology of human leptospirosis in Fiji.

**Methods:**

We obtained field data from a cross-sectional community survey done in 2013 in the three main islands of Fiji. A blood sample obtained from each participant (aged 1–90 years) was tested for anti-*Leptospira* antibodies and household locations were recorded using GPS receivers. We used GWLR to quantify the spatial variation in the relative importance of five environmental and sociodemographic covariates (cattle density, distance to river, poverty rate, residential setting [urban or rural], and maximum rainfall in the wettest month) on leptospirosis transmission in Fiji. We developed two models, one using GWLR and one with standard logistic regression; for each model, the dependent variable was the presence or absence of anti-*Leptospira* antibodies. GWLR results were compared with results obtained with standard logistic regression, and used to produce a predictive risk map and maps showing the spatial variation in odds ratios (OR) for each covariate.

**Findings:**

The dataset contained location information for 2046 participants from 1922 households representing 81 communities. The Aikaike information criterion value of the GWLR model was 1935·2 compared with 1254·2 for the standard logistic regression model, indicating that the GWLR model was more efficient. Both models produced similar OR for the covariates, but GWLR also detected spatial variation in the effect of each covariate. Maximum rainfall had the least variation across space (median OR 1·30, IQR 1·27–1·35), and distance to river varied the most (1·45, 1·35–2·05). The predictive risk map indicated that the highest risk was in the interior of Viti Levu, and the agricultural region and southern end of Vanua Levu.

**Interpretation:**

GWLR provided a valuable method for modelling spatial heterogeneity of covariates for leptospirosis infection and their relative importance over space. Results of GWLR could be used to inform more place-specific interventions, particularly for diseases with strong environmental or sociodemographic drivers of transmission.

**Funding:**

WHO, Australian National Health & Medical Research Council, University of Queensland, UK Medical Research Council, Chadwick Trust.

## Introduction

Leptospirosis is one of the most common bacterial zoonoses worldwide, causing more than one million severe infections each year.^1^, ^2^ Mammals including rodents, livestock, wildlife, and pets are the primary hosts for pathogenic *Leptospira*. Human infections occur through direct contact with infected animals, or contact with an environment that has been contaminated by the urine of infected animals. The transmission dynamics of leptospirosis are therefore complex and vary between places, depending on interactions between human beings, animals, and the environment, including occupational and recreational exposures.^3^, ^4^, ^5^, ^6^, ^7^ Unprecedented outbreaks have been increasingly reported from around the world; major drivers of the increased transmission include climate change and extreme weather events (particularly flooding), urbanisation, poverty, and agricultural intensification.^3^, ^6^, ^7^, ^8^ The intensity and relative importance of the environmental drivers vary between location type (eg, urban, periurban, and rural) and geographical scales (eg, communities, regions, and countries).

In the Pacific islands, leptospirosis is responsible for substantial morbidity and mortality, and the incidence is among the highest in the world.^1^ Major environmental drivers in the region include the tropical climate, cyclones, flooding, poor sanitation, subsistence farming, and the abundance of rodents.^9^ In 2012, leptospirosis outbreaks occurred after two consecutive severe floods in Fiji, with more than 500 reported cases and more than 50 deaths—a significant health burden for a small population.^6^ Subsequently, an eco-epidemiological study^10^ of leptospirosis was undertaken in Fiji to improve understanding of the behavioural and environmental factors associated with infection. Using multivariable regression modelling, the investigators identified individual behavioural and demographic risk factors including sex and ethnicity, and several socio-demographic and environmental covariates that were significantly associated with a higher probability of infection, including rural location, high poverty rate, high rainfall, close proximity to rivers, presence of pigs in the community, and high cattle density. Findings from the study also suggested that the probability of infection varied significantly between regions and residential settings, and between individual communities in each type of residential setting.

**Research in context**

**Evidence before this study**

Leptospirosis is one of the most common bacterial zoonotic diseases in the world, with significant knowledge gaps regarding its epidemiology, disease ecology, and transmission dynamics. The ability to design and implement effective prevention strategies has been restricted by the inability of tools to accurately identify, predict, and forecast hotspots of transmission. Sociodemographic and environmental drivers can vary significantly over geographical spaces and between socioecological niches (eg, urban *vs* rural areas, land use, sociodemographics, and ethnicity), even within the same country. Although non-spatial regression methods are unable to capture variations in these relationships over geographical space, geographically weighted logistic regression (GWLR) accounts for the spatial heterogeneity of relationships by allowing the coefficient of each covariate to vary over space. We searched PubMed on Jan 9, 2017, for studies published up to this date with the search terms “leptospirosis”, “*Leptospira*”, “map*”, “geographically weighted regression”, and “spatial non-stationarity”. We restricted the search to articles published in English, and did not identify any articles that used geographically weighted regression to model or map a combination of environmental and sociodemographic drivers of leptospirosis.

**Added value of this study**

We showed that GWLR and non-spatial, standard logistic regression models had equal power to predict hotspots of transmission (as measured by the average area under the receiver operating curve). However, GWLR was also useful for modelling spatial variation in the relationships between dependent and independent variables. Using GWLR, we identified significant geographical variation in the intensity of environmental and sociodemographic drivers, and provided new insights into leptospirosis transmission in Fiji. This study also added value to scientific literature on leptospirosis modelling and mapping by using GWLR to account for and demonstrate the spatial non-stationarity of covariates.

**Implications of all the available evidence**

Maps of the geographical variation in the odds ratios (ie, relative importance) of each covariate affecting leptospirosis provide a visually powerful way of conveying the findings to public health practitioners. The information can be used to develop more targeted public health interventions, and prioritise strategies that are most likely to be effective in different locations. The concepts and methods used in this study could also be applied to other diseases, particularly those with strong environmental and sociodemographic determinants.

Although standard multivariable regression methods provide good results when the strength and significance of the relationships between the dependent variable and the covariates are constant throughout the spatial distribution of the sample, they are not able to capture variations in these relationships over a geographical space. Geographically weighted regression is a spatial regression method that allows the coefficient of each covariate to vary over a geographical space.^11^ This method has been used in infectious disease epidemiological studies to investigate the spatial determinants of hand, foot, and mouth disease;^12^ assess the relationship between dengue incidence and socioeconomic parameters and population density;^13^, ^14^, ^15^ and to explore potential links between hydrological dynamics and human leptospirosis.^16^

Our study reported here built on previous findings^10^ by using geographically weighted logistic regression (GWLR) to identify and quantify the spatial variation in the relative importance of sociodemographic and environmental covariates on leptospirosis transmission in Fiji. The objectives of the study were to assess the performance of a GWLR model compared with a standard non-spatial logistic regression model; to determine if GWLR can provide additional insights into the eco-epidemiology of leptospirosis, particularly from a geographical perspective; and to identify potential public health interventions that were most likely to be effective in different parts of the country.

## Methods

### Study location and population

Fiji is an archipelago of 322 tropical islands located in the south Pacific with a population of 837 217.^13^ The two main ethnic groups are iTaukei (indigenous Fijians) and Indo-Fijians (Fijians of Indian descent), comprising 57% and 35% of the population, respectively. More than 90% of the population live on the three main islands of Viti Levu, Vanua Levu, and Taveuni in urban, periurban, and rural residential communities.^17^ The UN has classified Fiji as a small island developing state,^18^ because of its size and gross domestic product per person per year of US$4712.^19^ Fiji is divided into four divisions (Western, Central, Northern, and Eastern), which are further subdivided into 14 provinces and 86 tikinas, each comprising enumeration areas of 80 to 120 households.

### Sampling design and field survey

Detailed descriptions of the survey design and data collection have been previously reported.^6^ Briefly, field data were collected through a cross-sectional community survey in 2013, and sampling was designed to provide a study population that was representative of the communities on the three main islands of Fiji. For each participant, a blood sample was obtained for anti-*Leptospira* antibodies using the microscopic agglutination test, and household locations were recorded using handheld global positioning system (GPS) receivers. Ethics approvals in both Fiji and Australia were granted and each individual participant provided written informed consent.

The resulting dataset contained locations for 2152 participants from 1922 households. Of these households, 106 lacked accurate GPS coordinates and these data were excluded from further analyses. The records for the remaining 2046 participants represented 81 different communities, each including between five and 56 participants.

### Geospatial data and preparation

**Figure 1:**
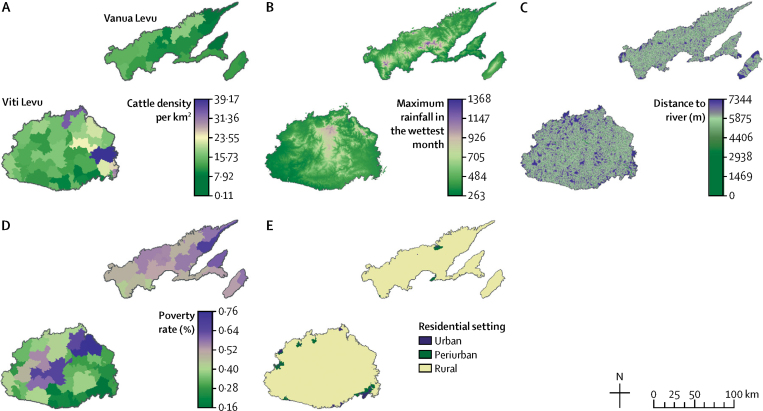
Spatial variation for five independent covariates associated with an increased probability of leptospirosis infection in Fiji Graphs show (A) cattle density per km^2^, (B) maximum rainfall in the wettest month (mm), (C) distance to river (m), (D) poverty rate, and (E) residential setting.

Data for covariates were obtained in georeferenced formats from several sources (table). On the basis of findings from the 2013 study of leptospirosis in Fiji,^10^ our analyses focused on five covariates: cattle density, maximum rainfall in the wettest month, distance to river, poverty rate, and residential setting (urban or rural; figure 1). The presence of pigs in the village (obtained from questionnaire data collected during the community survey) was found to be a significant covariate in the 2013 study, but was not included here because this information was only available for the 81 communities surveyed in 2013.

**Table:** Data sources and encoding methods for environmental and sociodemographic covariates

	**Description and spatial resolution**	**Encoding**	**Sources**
Cattle density	Density per sq km by Tikina (polygon)	Standardised to α=0 and σ=1	Fiji Ministry of Agriculture. Fiji National Agricultural Census 2009
Maximum rainfall	Average maximum annual rainfall, interpolated from meteorological data from 1971–2000; 100 m raster	Standardised to α=0 and σ=1	Landcare Research Institute, New Zealand^20^
Distance to river	Euclidean distance to river; 25 m raster data	Categorised as ≥100 m or <100 m	Hydrology data from Fiji Ministry of Lands and Mineral Resources 1:50K topographic maps^21^
Poverty rate	Percent of population below the poverty line by Tikina (polygon)	Categorised as <40% or ≥40% of population below the poverty line	World Bank (2011) report^22^
Residential setting	Census 2007 designations of urban, periurban, and rural by Enumeration Area (polygon)	Categorised as rural, urban, or periurban	Fiji Bureau of Statistics. Fiji National Census 2007^17^

A polygon lattice of hexagons (each measuring 200 m from side to side) was created to spatially standardise the data to a common geographical unit. Hexagons further than 1000 m from any inhabited areas were excluded from the model because data on sociodemographic variables were irrelevant or unavailable. For the two continuous raster datasets (maximum rainfall and distance to river), we used the mean value within each polygon. For the three vector datasets (cattle density, poverty rate, and residential setting), the most common value was used. For each of the 2046 datapoints, household GPS locations were overlaid onto each of the five hexagon layers to obtain covariate values at each location.

### Regression models

We built two logistic regression models, one using standard logistic regression, and another using GWLR. For each model, the dependent variable was the presence or absence of anti-*Leptopsira* antibodies, and the covariates examined were cattle density, maximum rainfall in the wettest month, distance to river, poverty rate, and residential setting (urban or rural).

The standard logistic regression model is a global model that does not account for spatial variation, and produces a single coefficient of determination (R^2^) and a single β coefficient for each covariate.^11^ By contrast, GWLR models the relationship between dependent and independent covariates as a series of local models. Each observation is modelled separately, and only includes other observations within a neighbourhood (kernel) based on a fixed distance, or an adaptive distance (based on sample point density) from the modelled observation where points closer to the regression point are weighted heavier than those further away.^23^ A GWLR model produces multiple coefficients of variation and β coefficients, one for each observation in the sample,^11^ and can be expressed formally as:

$$Ln\left(\frac{P_{i}}{1-P_{i}}\right)=\sum_{k}\beta_{k}(u_{i},v_{i})x_{k,i}+{ɛ}_{i}$$

Where:

$$Ln\left(\frac{P_{i}}{1-P_{i}}\right)$$

is the predicted odds for the *i*th observation, and *x*_ki_ and ɛ_i_ are, respectively, the *k*th covariate and error at observation *i*; (*u*_i_,*v*_i_) is the x,y location of the *i*th observation; and β_k_(*u*_i_,*v*_i_) the coefficient for *k*th independent variable for the observation at location *i*.^23^ For this study, we used an adaptive kernel that included the 1000 neighbouring points nearest to each regression point.

To compare performance between the two models, repeated random subsampling was used to create 50 trials for both models. For each trial, 50% of the data were used in the training set and the remaining 50% in the testing set. Random subsamples were generated from the georeferenced shapefiles in ArcGIS (version 10.3, ESRI, Redlands CA), and exported to text files to run trials in R^24^ for both logistic regression (using the stats package) and GWLR (using the GWmodel package^25^) models. Both models were assessed using the average area under the receiver operating curve (AUC) produced over 50 trials, and the Akaike information criteria (AIC) of the models created using the complete dataset. To visually present spatial non-stationarity of the covariates, which describes variations in relationships between the dependant variable and covariates over space,^26^ maps of the odds ratio (OR) for each covariate were produced.

### Predicted seroprevalence maps and hotspot analysis

Prediction maps of the probability of leptospirosis infection were created by mapping the results of the GWLR model for each hexagon in the lattice. A difference map was created to show the differences in predicted probabilities between the logistic regression and GWLR models across the study area. To test for statistically significant higher or lower probability of infection, a hotspot analysis was done on the mapped GWLR predicted seroprevalence using the Getis-Ord Gi* statistic^27^ with ArcGIS. The Gi* statistic is a *Z* score that, assessed against the normal probability distribution, identifies clusters of higher or lower values within the CI. A Getis-Ord Gi* analysis assesses the value at a location (eg, probability of infection at a hexagon) in relation to values at locations within a surrounding neighbourhood to produce the Gi* statistic. Using the default settings, ArcGIS provided a suitable neighbourhood distance of 639 m.

### Role of the funding source

The funders of the study had no role in study design, data collection, data analysis, data interpretation, or writing of the report. The corresponding author had full access to all the data in the study and had final responsibility for the decision to submit for publication.

## Results

When run over the entire dataset, AIC values for the logistic regression and GWLR models were 1935·2 and 1254·2 respectively, indicating that the GWLR model was more efficient. Over 50 trials, the logistic regression and GWLR models both had a mean AUC of 0·64 (SD 0·016).

**Figure 2:**
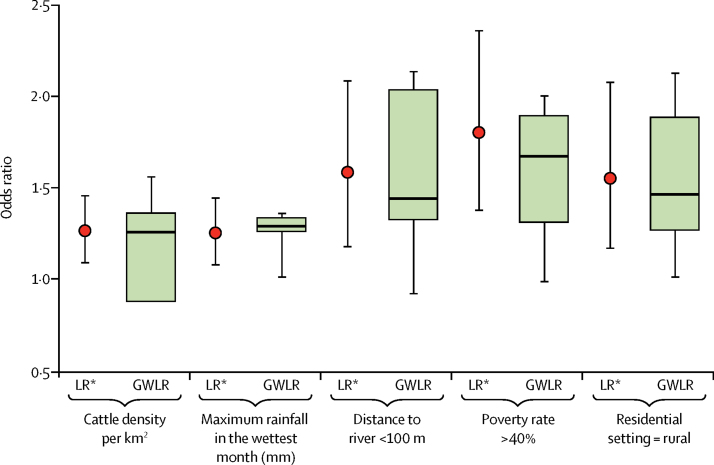
Logistic regression (LR) and geographically weighted logistic regression (GWLR) models for each covariate For the logistic regression model, the mean odds ratios and 95% CI are presented. For the GWLR model, the odds ratios are shown as median, IQR, minimum, maximum, and range. Variations in the odds ratios in the GWLR model indicate spatial variation in the effect of each covariate.

Maximum rainfall in the wettest month had the least variation in effect across the study area (GWLR median odds ratio [OR] 1·30, IQR 1·27–1·35; logistic regression OR 1·26, 95% CI 1·09–1·45), and distance to river had the greatest (GWLR median OR 1·45, IQR 1·35–2·05; logistic regression OR 1·61, 95% CI 1·24–2·18; figure 2). The differences between the upper and lower 95% CIs for the each covariate in the logistic regression model, and the range in OR for the covariates in the GWLR model were highly correlated (Pearson correlation coefficient 0·90), suggesting that a significant component of the imprecision in the standard logistic regression model was captured by the GWLR as geographical variation in the effect of the covariates.

**Figure 3:**
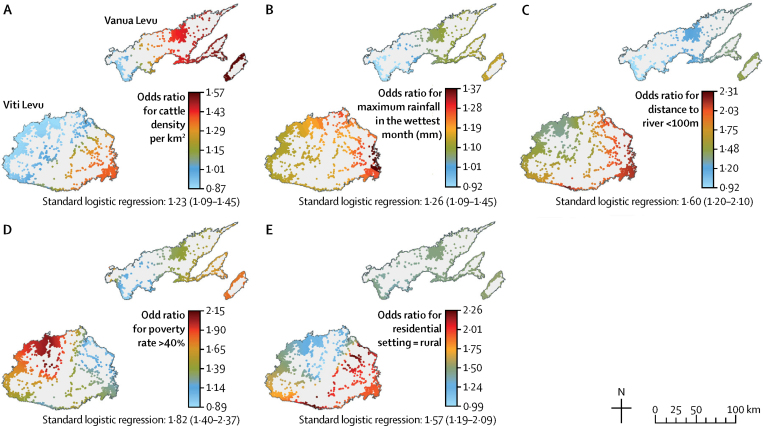
Spatial variation in the influence of each covariate calculated by the model Data are expressed as odds ratios (OR) for (A) cattle density per km^2^, (B) maximum rainfall (mm), (C) distance to river <100 m, (D) poverty rate >40%, and (E) rural residential settings. OR calculated by the logistic regression model and 95% CI are provided. A higher OR for a variable indicates that it has more influence in that area.

Substantial spatial variation in OR for each covariate was detected (figure 3). For example, the ORs for distance to river ranged from 2·31 in eastern Viti Levu and other areas to 0·92 in southwestern Vanua Levu.

**Figure 4:**
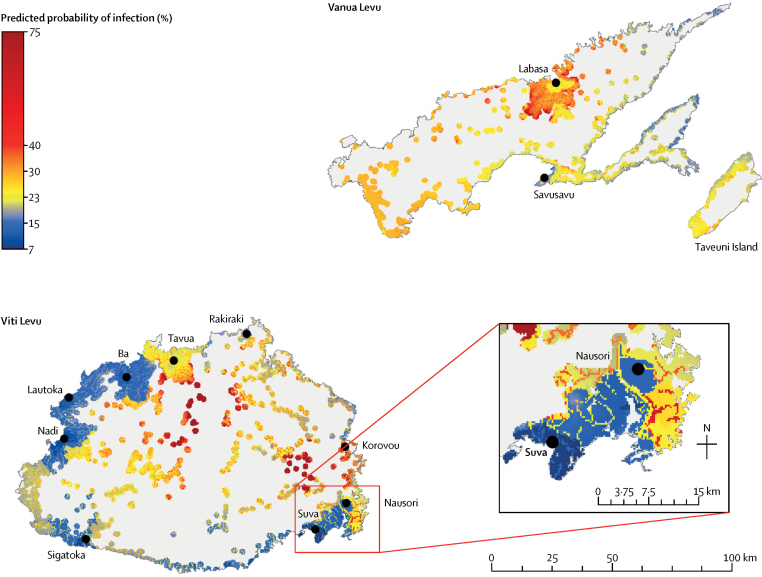
Model predictions of the probability of leptospirosis infection in Fiji on the main Islands of Viti Levu, Vanua Levu, and Taveuni Overall seroprevalence of anti-*Leptospira* antibodies was 20% in the 2013 study.

The GWLR model was used to produce a map of the predicted probability of leptospirosis infection (figure 4). The map indicates the highest risks were in the interior of Viti Levu, the agricultural region around Labasa on Vanua Levu, and the southern end of Vanua Levu. The model predicted lower risks in the urban areas of Suva, Sigatoka, and Nadi on Viti Levu.

**Figure 5:**
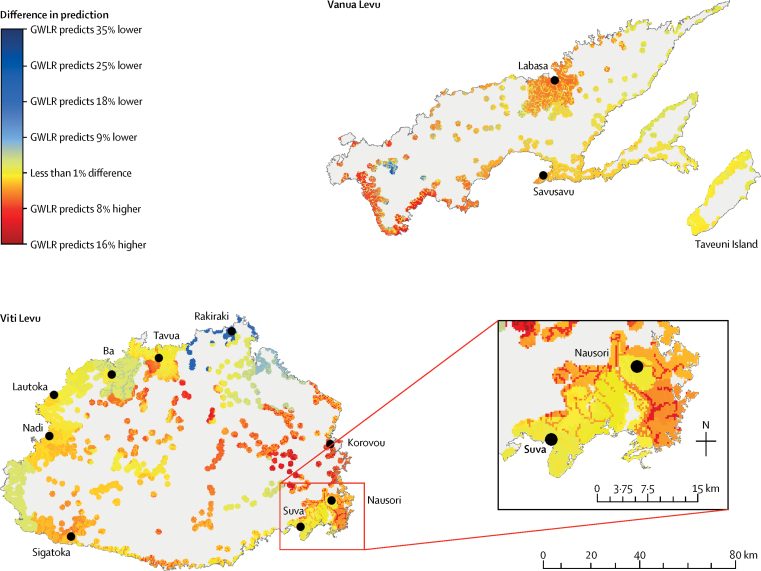
Spatial variation in differences in predicted probability of leptospirosis infection between logistic regression and GWLR models Graphs indicate areas where geographically weighted logistic regression (GWLR) predicted a higher or lower probability of infection compared with logistic regression. GWLR predicted the same or higher probability as the logistic regression model, with exceptions around Rakiraki and a small section in the east of Vanua Levu.

The differences between the predicted probabilities of infection estimated by the models are in figure 5. Taking the global average of ORs as determined by the logistic regression model, the map showed that GWLR predicted a higher probability of infection in some areas (eg, the interior of Viti Levu and around Labasa) and a lower probability in others (eg, around Ba, Rakiraki, and the southeast coast of Viti Levu).

**Figure 6:**
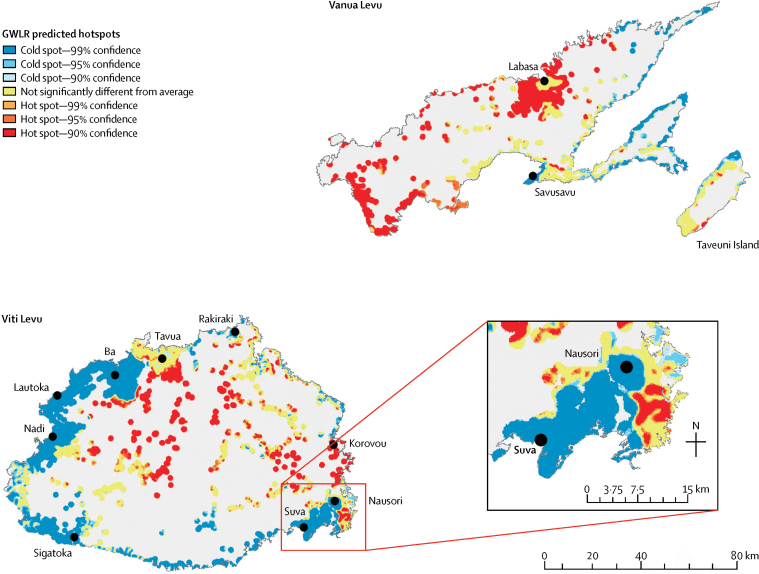
Cluster analysis showing areas predicted by geographically weighted logistic regression (GWLR) model as having significantly higher (hot spot) or lower (cold spot) than average probability of leptospirosis infection (19%) Created using Getis-Ord Gi* analysis. Cold spots indicated significantly less chance of infection around the urban areas of Suva and in the southwest of Vitu Levu, and in the east of Vanua Levu and northen Taveuni.

The Getis-Ord Gi* hotspot analysis presents the spatial distribution of probabilities from the GWLR model as being significantly higher or lower than the global mean prediction with CIs of 90%, 95% and 99% (figure 6). Predicted hotspots are within the interior of Viti Levu, around Labasa, and the southern point of Vanua Levu.

## Discussion

Our study showed that both standard logistical regression and GWLR models had similar predictive capability (based on the AUC), but GWLR was more efficient (based on AIC) and identified geographical variation in the intensity of sociodemographic and environmental drivers of leptospirosis transmission in Fiji. These results corroborate findings from other studies showing that geographically weighted regression can offer improvements and additional insights over standard non-spatial regression models for eco-epidemiological studies of infectious diseases and public health.^15^, ^16^, ^28^ The ORs for the logistic regression model were similar to the median ORs for the GWLR model, which was as expected considering that the results of the logistic regression model represented a global average for the study area.

The map of predicted probabilities of infection risk showed spatial variation in the differences and illustrated that including local geographical variation (non-stationary processes) in the modelling affected predicted probabilities. The average probability of infection is approximately 20%,^10^ and below average (depicted as cold spots in our map) does not necessarily mean that the probability of infection was negligible. The AIC, which assesses the relative quality of models given trade-offs between model fit and model complexity,^29^ showed that the GWLR model was more efficient than the standard logistical regression model, and the reduction in AIC in this study was substantial compared to similar GWLR studies, which have reported improvements in AIC of, at the most, around 200.^15^, ^16^ AIC can be thought of as a measure of how well a model uses information or how efficient it is. The lower AIC for the GWLR model suggested that by incorporating spatial information and modelling spatial non-stationarity, this model performed more efficiently.

Based on the AUC results, which provided a measure of predictive capability, both models performed equally. The low average AUC for both models indicated that covariates other than those modelled in this study also contributed to the probability of infection. Since many known risk factors for leptospirosis are related to individual-level variables (eg, sex, age, ethnicity, and behaviour), this finding was not unexpected.^10^ The AUC results were lower than the value of 0·7 reported in the 2013 study^10^ that used a standard logistical regression model, most likely because the covariate of presence of pigs in the community was not included in this study. Data for this variable (collected using questionnaires during the 2013 field study) were only available for the 81 communities included in the survey, and reliable country-level data on pig density were not readily available.

A comparison between the ORs and 95% CI for the logistical regression model and the median and range of ORs for the GWLR suggested that fundamentally, both models captured similar associations between leptospirosis and each covariate. The main advantage of GWLR is that it explicitly captures geographical variation of model coefficients by modelling spatial non-stationarity, whereas the logistical regression model considers this variation as a model error, expressed through wider CIs. In doing so, the GWLR offered a meaningful way to analyse and observe geographical variation in ORs for each covariate.

There are several possible explanations for the geographical variation in the relative importance of covariates. First, the values for each covariate varied geographically; eg, cattle density was higher in eastern Viti Levu and northeast Vanua Levu, which could partly explain the higher ORs for cattle density in those areas. Comparing the geographical distribution of covariates with the odds of infection suggests that this explanation holds true for some covariates such as maximum rainfall in the wettest month; the heaviest concentration of high rainfall was in the northern and interior mountainous region of Viti Levu, and high rainfall was associated with a stronger effect on the probability of infection in this region. Poverty rate, however, does not fit this explanation; it was highest in Vanua Levu and the interior of Viti Levu, yet its effect on probability of infection was strongest in northwest Viti Levu. This observation showed that GWLR results were not simply a reflection of the geographical distribution of the covariates, but provided deeper insights into the relative importance of the drivers of transmission.

Using maps to show the geographical variation in the relative importance of each covariate provides a visually powerful way of conveying the findings of a GWLR model to public health practitioners, who could use this knowledge to identify the most important drivers of transmission at different locations, and prioritise and more precisely target place-specific public health interventions. In a developing country such as Fiji where resources are limited, improving the cost-effectiveness of interventions is particularly important. For example, in areas where cattle density is strongly associated with leptospirosis (eg, high ORs in Vanua Levu and Taveuni), interventions related to reducing exposure to livestock and improving animal husbandry practices could be prioritised. Similarly, in areas where infection is more strongly associated with rainfall, flood mitigation and improvements in drainage are more likely to be effective compared with similar interventions in areas with a low OR for rainfall. Of note, in Ba (northwest Viti Levu), where the most severe post-flooding outbreaks occurred in 2012, the GWLR model showed strong association between maximum rainfall in the wettest month (a proxy measure for flooding risk) and leptospirosis.

Our results should be interpreted in light of the study's limitations. First, the models only included five covariates; however, we focused on these because they represented five of the six significant sociodemo-graphic and environmental covariates identified in the 2013 study,^10^ and suitable country-level georeferenced data were available. The predictive capability of our models could be improved by including other spatially explicit covariates, but for this modelling framework it was not possible to include environmental or sociodemographic factors for which country-level georeferenced data were not available. The 2013 study also identified significant individual-level covariates (eg, sex and behaviour); it is not practical to include such covariates as spatial layers but it is possible to produce separate maps for subpopulations, as shown in a study of leptospirosis in American Samoa.^5^

Another limitation is the modifiable areal unit problem,^30^ which states that the level of geographical aggregation of spatial phenomena is a source of bias for statistical hypothesis tests. Using a lattice of 200 m hexagons as the modelling framework was a source of bias; the parameter estimates from our models might have differed if different sized hexagons or different geographic units had been used. However, it is unusual for all georeferenced data from several sources to be of the same scale and resolution, so spatial standardisation is generally necessary.

Finally, regression models determine association rather than causation. For example, people living in areas with a high poverty rate are also more likely to have poor sanitation, poor access to clean water, more intense exposure to subsistence livestock, more likely to be involved in outdoor occupations such as farming, and more likely to bathe or swim in rivers. These factors are known to be associated with a higher risk of leptospirosis infection, and a statistical association between poverty and leptospirosis does not help to determine the most likely causal pathway(s).

This study showed the value of GWLR in improving understanding of the environmental and sociodemo-graphic drivers of infectious disease transmission, and the importance of considering the spatial heterogeneity of covariates as well as their relative importance over space. A key advantage of GWLR is the ability to visually present useful information about the importance of covariates on the dependent variable in a way that captures non-stationarity in the relationships. The outputs of the GWLR model could then be assessed for significant clusters using Getis-Ord Gi* to visually identify geographical hotspots of leptospirosis transmission.

Significant geographical variations in the relative importance of environmental and sociodemographic drivers were noted even within the islands of Fiji, which are very small. Spatial non-stationarity could therefore be more important in studies involving larger and environmentally more diverse areas, where covariates might vary more dramatically. GWLR provided important insights into the drivers of leptospirosis transmission, and valuable information and maps to inform more effective and cost-efficient interventions. Although the results of this study are specific to leptospirosis in Fiji, the concepts and methods can be applied to improve understanding of the ecoepidemiology of other infectious diseases or other settings and inform the design and implementation of place-specific interventions. Understanding the spatial variation in the relative importance of risk factors is particularly valuable for infectious diseases with strong environmental and sociodemographic determinants, which are typically spatially heterogeneous. The results of this study are specific for leptospirosis in Fiji, but the concepts and methods could be applied to other infectious diseases and other locations, particularly for diseases with strong environmental and sociodemographic drivers of transmission.
